# Techniques to Study Inflammasome Activation and Inhibition by Small Molecules

**DOI:** 10.3390/molecules26061704

**Published:** 2021-03-18

**Authors:** Diego Angosto-Bazarra, Cristina Molina-López, Alejandro Peñín-Franch, Laura Hurtado-Navarro, Pablo Pelegrín

**Affiliations:** Línea de Inflamación Molecular, Instituto Murciano de Investigación Biosanitaria IMIB-Arrixaca, Hospital Clínico Universitario Virgen de la Arrixaca, 30120 Murcia, Spain; cristina.molina@imib.es (C.M.-L.); alejandroe.penin@imib.es (A.P.-F.); laura.hurtado1@um.es (L.H.-N.)

**Keywords:** inflammasomes, small molecules, techniques to assay inflammasome activation, auto-active inflammasomes, NLRP3 inhibitors

## Abstract

Inflammasomes are immune cytosolic oligomers involved in the initiation and progression of multiple pathologies and diseases. The tight regulation of these immune sensors is necessary to control an optimal inflammatory response and recover organism homeostasis. Prolonged activation of inflammasomes result in the development of chronic inflammatory diseases, and the use of small drug-like inhibitory molecules are emerging as promising anti-inflammatory therapies. Different aspects have to be taken in consideration when designing inflammasome inhibitors. This review summarizes the different techniques that can be used to study the mechanism of action of potential inflammasome inhibitory molecules.

## 1. Introduction

**Figure 1:**
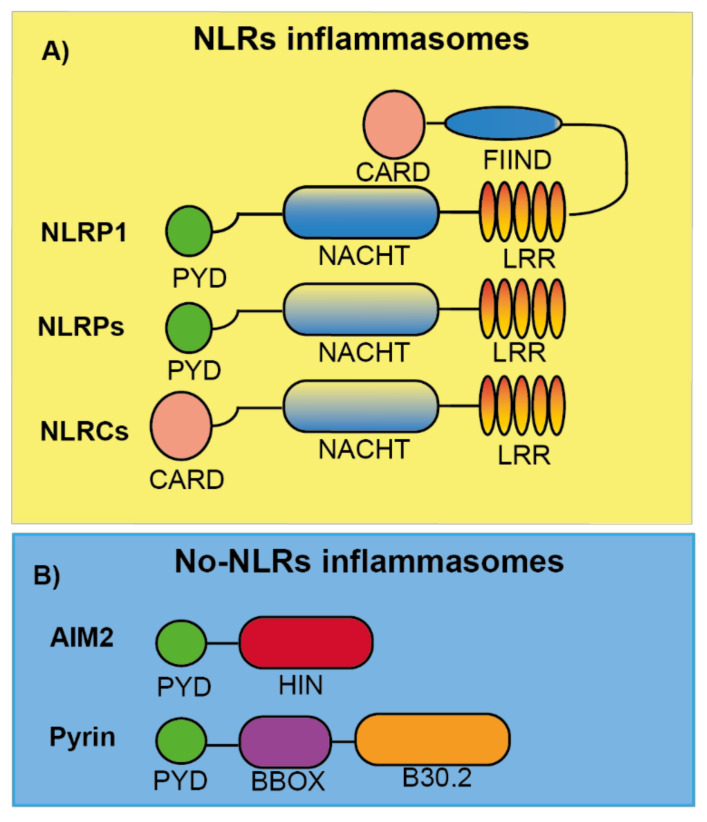
Schematic representation of the different inflammasome sensor proteins. (**A**) Nod like receptor (NLR) proteins are formed by either a PYD (NLRP) or CARD (NLRC) domain at the N-terminal position follow by a central domain (NACHT), responsible for NLR oligomerization, and a C-terminal leucine-rich repeat domain (LRR). For NLRP1, a FIIND and CARD domain are in the C-terminal position after the LRR domain. (**B**) AIM2 and Pyrin are no-NLR inflammasome sensors with a PYD domain at the N-terminal. AIM2 also presents a HIN domain responsible of DNA binding. Pyrin is also formed by a central coiled BBOX domain and a C-terminal B30.2/SPRY domain.

Nearly two decades ago, an intracellular caspase-activating multiprotein complex was named as the “inflammasome” [1]. Inflammasomes can be defined as cytosolic-multimeric-high molecular weight protein oligomers that are formed in response to different intrinsic and/or external cellular stimuli including pathogen-associated molecular patterns (PAMPs), damage-associated molecular patterns (DAMPs) or homeostasis-altering molecular processes (HAMPs) (Table 1) [2,3,4]. The inflammasomes are classified and named according to the sensor protein that triggers its activation, the different inflammasome sensors share structural motifs and could belong to the nucleotide-binding domain and leucine-rich-containing family of receptors (NLRs), the absent in melanoma 2-like receptors (ALRs) and pyrin (Figure 1) [5,6].

**Figure 2:**
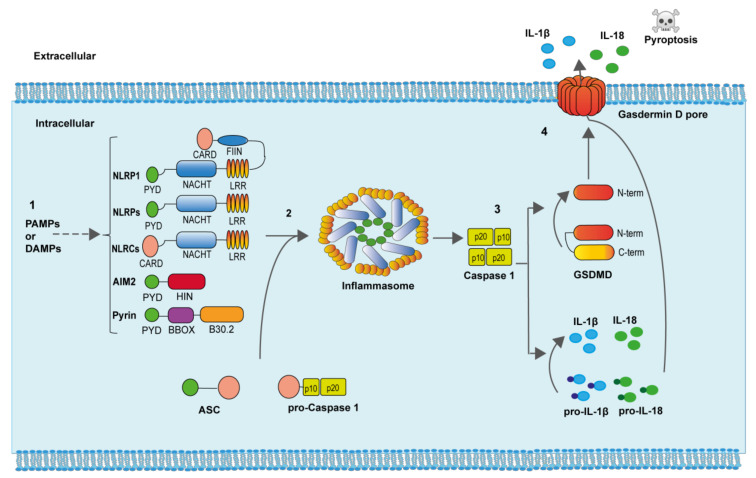
Inflammasome activation. (**1**) Specific PAMPs and DAMPs are able to induce both the expression and oligomerization of the different inflammasome sensor proteins. (**2**) The adaptor ASC protein oligomerizes within the inflammasome and recruits pro-caspase-1, favoring caspase-1 autoactivation. (**3**) Active caspase-1 cleaves the precursors of the pro-inflammatory cytokines IL-1β and IL-18, as well as the protein GSDMD. The N-terminal portion of GSDMD binds and produce pores in the plasma membrane. (**4**) The active pro-inflammatory cytokines IL-1β and IL-18, as well as diverse intracellular content, is released through GSDMD pores. Increasing formation of GSDMD pores produces cell swelling and pyroptotic cell death.

To engage the inflammasomes, these sensors homo-oligomerize and directly recruit the zymogen of caspase-1 through homotypic interactions or through the adaptor molecule apoptosis-associated speck-like protein containing a caspase recruitment and activation domain (ASC) as occurs in most of the inflammasomes. ASC oligomerization to inflammasome sensors result in one of the hallmarks of inflammasome activation, the formation of a large ASC oligomer named “ASC speck” [7,8]. The zymogen of caspase-1 is activated within the inflammasome due to proximity induced autocatalytic cleavage of two precursors subunits [9]. Once fully activated, caspase-1 cleaves different protein substrates in the cell that include the consensus sequence YVHD/FESD [10]. Among the different caspase-1 substrates, we found the pro-inflammatory cytokines interleukin (IL)-1β and IL-18, and the protein gasdermin D (GSDMD) [11]. After caspase-1 processing, the generated N-terminal fragment of GSDMD binds to negatively charge lipids of the plasma membrane inner bilayer and oligomerizes producing a pore of about 10-12 nm, from where the mature IL-1β and IL-18, as well as other intracellular content, is released from the cell [11]. If GSDMD pores are not efficiently removed from the plasma membrane, a specific type of cell death, called pyroptosis, is produced (Figure 2) [11,12].

Inflammasomes are important components of host defense and are able to induce an effective immune response. A controlled activation of inflammasomes is critical to obtain an optimal response against different insults, including infections. A dysregulation of inflammasome activation is associated with different pathologies, such as neurodegenerative diseases (like Alzheimer or multiple sclerosis), metabolic diseases (like type 2 diabetes or non-alcoholic steatohepatitis), autoimmune diseases (like rheumatoid arthritis or gout), the rejection of allogeneic transplants, and autoinflammatory syndromes where specific mutations in different inflammasome sensors encoding genes are responsible of an exacerbated inflammasome activation [13,14,15,16,17,18,19,20,21]. Due to the important implication of the inflammasomes in different pathologies, the identification of specific inflammasome inhibitors is a promising growing field to manage different diseases that currently lack effective therapies [22]. Different techniques are needed to reveal the mechanism of action of inflammasome inhibitors, and this review aims to summarize them.

## 2. NLR Inflammasomes

NLR inflammasomes can be grouped into two major families, the NLRPs and the NLRCs, depending on whether the N-terminal presents a pyrin (PYD) or caspase recruitment and activation (CARD) domain, respectively (Figure 1A) [6]. In humans, there are 14 NLRP and 5 NLRC identified [23]. Both share some features as a C-terminal leucine-rich repeat domain (LRR) and a central domain responsible for NLR oligomerization found in different plant and animal proteins named NACHT (neuronal apoptosis inhibitory protein NAIP, major histocompatibility class II transcription activator CIITA, incompatibility locus protein from *Podospora anserina* HET-E, and telomerase-associated protein TP1) [23]. Several proteins of this family have been well characterized to form inflammasomes, among them we found the NLRP1, NLRP3, NLRP6, and NLRC4 inflammasomes, while other NLRs members remain to be studied if they are able to form inflammasomes in response to specific stimuli.

### 2.1. NLRP1 Inflammasome

NLRP1 was the first inflammasome to be described [1]. The NLRP1 has different domains: PYD, NACHT, LRR, a function to find (FIIND) domain, and a CARD domain (Figure 1A). In mice, NLRP1 presents three different isoforms: NLRP1a, NLRP1b, and NLRP1c, and differentially from the single human NLRP1, all of them lack the PYD domain. NLRP1b has been widely studied and is activated by anthrax lethal toxin that is produced by *Bacillus anthracis*. Anthrax lethal toxin is formed by a protective antigen and a lethal factor with metalloprotease activity responsible for the specific cleavage inside the FIIND domain of NLRP1b. This cleavage is necessary to activate the NLRP1 inflammasome, since the NLRP1b N-terminal fragment is degraded by the proteasome, liberating the C-terminal CARD domain responsible for caspase-1 activation [24,25,26,27] (Table 2). Therefore, the NLRP1b needs the processing of its FIIND domain to produce an active inflammasome, either by external stimulations or autoprocessing [28]. NLRP1b activation is important for parasite clearance and acquiring specific protection against *Toxoplasma gondii* [29]. This role has also been found for the human NLRP1, where a loss of NLRP1 in monocytic cells fosters *Toxoplasma* infectivity [30]. However, the specific ligand or activation for the human NLRP1 activation has not been elucidated yet [31]. In humans, specific mutations of *NLRP1* gene are related with vitiligo, Addison’s disease, skin inflammation, and cancer susceptibility [32,33,34], but more studies are necessary to shed light into the molecular mechanism of NLRP1 induced pathology. So far, there are not any reported NLRP1 specific inhibitors that could be important to treat the impact of the diseases where NLRP1 is involved.

### 2.2. NLRP3 Inflammasome

This is the most studied inflammasome due to the wide number of pathologies where it is implicated. NLRP3 is formed by a PYD, NACHT, and LRR domain (Figure 1A). Many different studies have shown how NLRP3 can be activated by different triggers, suggesting the ubiquitous activation and importance of the NLRP3 inflammasome. NLRP3 can be activated by multiple infectious and endogenous ligands (Table 2), resulting in the most promiscuous of the inflammasomes. The different triggers of NLRP3 converges in some common cellular signaling as the decrease of the concentration of intracellular K^+^ (either by the activation of selective K^+^-conductance channels or plasma membrane permeabilization), production of reactive oxygen species (ROS), phagolysosome destabilization and the presence of oxidized mitochondrial DNA in the cytosol [35,36,37,38]. Therefore, NLRP3 can be considered as a sensor of the balanced intracellular environment, and some authors have suggested that NLRP3 is a sensor of HAMPs.

The canonical activation of NLRP3 in macrophages follow a two-step mechanism, where the first step is a priming of NLRP3 that is commonly achieved via Toll-like receptor (TLR) activation of nuclear factor kappa B (NF-κB). This priming results in the upregulation of *NLRP3* gene expression and induction of different post-transcriptional modifications of NLRP3, such as de-ubiquitination or phosphorylation/dephosphorylation events among others [39]. This priming step could be also triggered by cytokines as tumor necrosis factor (TNF)-α or IL-1. The initial priming step also increases the expression of pro-IL-1β in macrophages. The second activation step of NLRP3 induces the homo-oligomerization of NLRP3 and is induced by a wide variety of different triggers (Table 2). NLRP3 oligomers recruit and oligomerize the adaptor protein ASC via PYD/PYD homotypic interactions and finally ASC recruit caspase-1 to promote its activation within the inflammasome.

Multiple other proteins can also bind to NLRP3 and are necessary for the activation of this inflammasome. Among them, never in mitosis gene a (NIMA)-related kinase 7 (NEK7), a member of the NIMA related kinases, has an important role in NLRP3 activation [40,41]. NEK7 acts downstream of the K^+^ efflux regulating the activation of NLRP3 by a direct binding to the NACHT and LRR domains [40]. However, NEK7 implication in NLRP3 activation can be bypassed by TAK1-dependent post-translational priming [42], suggesting that the activation mechanism of NLRP3 inflammasome can be tangled. Post-translational modifications of NLRP3 during the priming step, as different phosphorylation events in the PYD and NACHT domains [43,44,45,46], are important for a correct NLRP3 activation. In addition, interaction of NLRP3 with negatively charged lipids as the phosphatidylinositol-4-phophate on dispersed trans-Golgi network is necessary for its activation [47], confirming that the mechanism of NLRP3 activation is a complex pathway not fully understood yet. NLRP3 inflammasome can also be activated in a non-canonical manner after caspase-4/5 (in humans) or caspase-11 (in mouse) activation triggered by intracellular lipopolysaccharide (LPS) recognition. Caspase-4/5/11 activation cleaves GSDMD and N-terminal GSDMD pore formation induces K^+^ efflux and the non-canonical activation of the NLRP3 inflammasome [42].

NLRP3 has an important role in different pathologies, since the aberrant activation of this inflammasome is associated with several conditions, such as arthritis, gout, diabetes, Alzheimer’s disease or obesity [6]. Specific mutations in the *NLRP3* gene have been associated with the development of a specific type of autoinflammatory manifestation termed cryopyrin-associated periodic syndromes (CAPS). CAPS individuals develop periodic skin rashes and fever [48], and the severity of these symptoms classify CAPS patients into: Neonatal-onset multisystem inflammatory disease (NOMID) as the most severe syndrome, Muckle-Wells syndrome (MWS) with intermediate symptoms, and the familial cold autoinflammatory syndrome (FCAS) as the milder form of CAPS triggered by low temperatures.

The fact that NLRP3 is involved in the initiation and progression of several human diseases lacking effective therapies has resulted in major advances on the understanding of NLRP3 activation mechanism and the development of several specific small molecules blocking NLRP3. However, we still do not fully understand the NLRP3 activation process, the mechanism of action of some NLRP3 blocking molecules, and their efficacy as novel drugs for humans.

### 2.3. NLRC4 Inflammasome

NLRC4 consists of a CARD, NACHT, and LRR domains (Figure 1A). The implication of ASC in this inflammasome is ambiguous because NLRC4 could directly promote caspase-1 activation, however, ASC seems necessary for IL-1β release [49], but not for pyroptosis [50] when NLRC4 is activated. NLRC4 does not directly bind triggering ligands as flagellin (Table 2), but there are proteins that belong to the NLR family of apoptosis inhibitory proteins (NAIPs) that recognize ligands and induce NLRC4 oligomerization. In mice, there are four different NAIPs, NAIP1 and NAIP2 recognize the bacterial needle and inner rod proteins, and NAIP5 and NAIP6 bind to flagellin [51,52,53,54]. Only one NAIP is present in humans, and this NAIP is able to sense the different NLRC4 activators [52,53,54]. The phosphorylation of Ser533 is important for NLRC4 activation after *Salmonella* infection, because it probably drives a conformational change in NLRC4 important for its activation [55]. Therefore, similarly to NLRP3, post-translational modifications in NLRC4 are needed for its activation.

Gain-of-function mutations in *NLCR4* gene have been associated with autoinflammatory diseases [17,56,57,58], with symptoms overlapping CAPS and in the most severe syndromes auto-active NLRC4 mutations associate with the macrophage activating syndrome (MAS). Differently from CAPS, the macrophage activating syndrome is mainly mediated by IL-18 and treatment with recombinant IL-18 binding protein has been successfully used in patients [56]. The design of specific inhibitors blocking NLRC4 is an important area of development, however, no small molecules blocking NLRC4 have been described so far.

### 2.4. NLRP6 Inflammasome

NLRP6 is an inflammasome with important roles as immunosensor. Its structure consists of a PYD, NACHT, and LRR domains (Figure 1A), but even sharing a 43.8% of similarity with NLRP3, NLRP6 cannot be activated by the same stimuli that NLRP3 senses (Table 2). The mechanism of activation and action of NLRP6 has not been well described to date, however it has been reported to form an inflammasome by interacting with ASC, but this results in caspase-11 activation instead of caspase-1 [59]. The PYD domain from NLRP6 is able to self-assemble in a filament manner that can bind ASC [60]. Most of the studies about the NLRP6 inflammasome have been focused in cells of the intestine, where it is highly expressed, and one of the main physiological roles of NLRP6 is to maintain the intestinal mucosal homeostasis in an optimal state [61,62]. Other physiological functions of NLRP6 have been described, including the inhibition in carcinogenesis and promoting neuroinflammation [63,64]. To date, no pathological mutations have been associated with NLRP6, and no specific inhibitors have been designed for this inflammasome.

## 3. Non-NLR Inflammasomes

### 3.1. AIM2 Inflammasome

AIM2 is the only member of the AIM2-like receptor (ALR) family that has been described to form an inflammasome. AIM2 presents a PYD and a HIN domain (Figure 1B), this last one is a domain that directly binds dsDNA (Table 2), dsDNA being a potent activator of this inflammasome [65]. The binding of AIM2 to dsDNA is independent of the origin or sequence of the DNA, although a length larger than 80 bp is needed to obtain a robust AIM2 activation [4]. The fact that AIM2 does not have a CARD domain makes the requirement of ASC necessary for the activation of caspase-1 within this inflammasome [66]. Other protein partners have been described to be required for AIM2 activation, and for example during *Francisella novocida* infection, the presence of guanylate binding proteins (GBP), specifically GBP2 and GBP5, are necessary to activate AIM2 [67].

Physiologically, AIM2 is considered as a therapeutic target for autoimmune disorders, specifically in psoriasis and systemic lupus erythematosus [68,69] where endogenous dsDNA is a trigger of these conditions. Additionally, an interesting role for AIM2 in cancer therapeutics has been described, where AIM2 mediates the caspase-1 dependent death of intestinal epithelial and bone marrow cells in response to dsDNA breaks produced by radiotherapy and chemotherapeutic agents, without affecting the anti-cancer properties of these drugs. This suggests that AIM2 could be modulated to decrease or limit the effects of chemotherapy [70].

### 3.2. Pyrin Inflammasome

Pyrin protein is formed by a PYD, a central coiled BBOX domain and a C-terminal B30.2/SPRY domain (SPia and the Ryanodine Receptor domain) (Figure 1B), this last domain is not present in the mouse Pyrin orthologous proteins. Unlike other inflammasomes, Pyrin is activated by pathogen-mediated modifications of host proteins (Table 2) and once is activated, ASC binds to the PYD domain to form an inflammasome, which activates caspase-1, IL-1β release, and GSDMD-dependent pyroptosis [71,72]. The protein 14-3-3 is important for Pyrin activation, as its binding to the phosphorylated Ser242 residue in Pyrin keeps this inflammasome inhibited. Pyrin dephosphorylation induced by small GTPases inhibition releases Pyrin from 14-3-3 and promotes Pyrin interaction with ASC [73]. Different bacterial toxins, as *Clostridium difficile* toxin B (TcdB) are potent inhibitors of GTPases, and therefore strong activators of the Pyrin inflammasome [74].

Pyrin inflammasome is important for the host response during bacterial infections and the deficiency of Pyrin increase bacterial load in macrophages [75]. Mutations in Pyrin have been associated with autoinflammatory human diseases, in particular to Familial Mediterranean fever [76], but also to myalgia, serositis, amyloidosis, and neutrophilic dermatosis [77]. Colchicine is the first line of drug used to treat familial Mediterranean fever, and anti-IL-1 therapy is used in case the patient is resistant to colchicine. However, any of these compounds directly block Pyrin inflammasome, therefore there is an urgent need to find specific Pyrin blockers.

## 4. Techniques to Measure Inflammasome Activation

To tackle the study of the mechanism of action of inhibitors for different inflammasomes is necessary to perform different cellular, molecular, and biochemistry techniques. The data obtained from these techniques will give important information about the mechanism of action of the inhibitor and will allow its future development towards clinics. Due to the high number of techniques used to assess the activation of the inflammasomes, in this review we will focus on the techniques that could be scaled up to do high throughput screenings and the ones to depict the mechanism of action of the selected molecules.

### 4.1. Determination of the Expression of Key Inflammasome-Associated Gene

The amount of mRNA detected by techniques such as quantitative polymerase chain reaction (qPCR) is relevant to those inflammasomes that requires a priming step related to an increase of the expression of the inflammasome sensor, as, for example, for NLRP3. However, other inflammasomes that do not require such priming, as Pyrin or AIM2, to assess if a possible inhibitor affects its expression will be less relevant. Furthermore, the modulation of the inflammasome sensor expression by small molecules is not going to be a direct proof that the final activity of such inflammasome is affected. In any case, it is relevant to assess if the small molecule in development affects the expression of the different inflammasome components, and therefore qPCR data is going to give valuable information about a possible mechanism of action. Expression of the inflammasome sensor and other inducible pro-inflammatory cytokines as pro-IL-1β is a routine check for potential compounds blocking the inflammasomes. The expression of other inflammasome components such as ASC, caspase-1, or gasdermin D could be also checked, but usually they are expressed at basal level and are not affected during inflammasome priming.

If a small molecule targeting an inflammasome decreases the expression of the inflammasome sensor or pro-IL-1β mRNA, that could suggest an inhibition of the priming step. Cell priming is usually achieved via TLR triggering, therefore the molecule could be affecting the TLR signaling or the activation of the transcription factor NF-κB. It is possible that a certain inhibitor could affect both the TLR signaling and also inflammasome activation, therefore its anti-inflammatory action could be broader in a physiological context, but it will not be specific for the inflammasome. If the inhibitor does not block the priming step and the expression of inflammasome sensor, associated proteins and pro-IL-1β are not affected, and then the molecule could have a chance to block the inflammasome at other level.

The determination of the mRNA expression for other inflammatory cytokines that are independent of the inflammasome, such as IL-6 or TNFα, is important to validate if the molecule is specific for the inflammasome priming, or goes beyond. The expression of inflammasome-independent cytokines should not be affected by an inhibitor targeting an inflammasome.

When performing qPCR, the selection of the housekeeping gene to normalize the expression of the different inflammasome-related genes is important. Housekeeping gene expression must be stable in resting conditions, after the priming of the cells, and in the presence of the small inhibitory molecules. If any of these conditions affects the expression of the housekeeping gene, an alternative reference gene should be selected. For example, the metabolic state of the macrophage changes during LPS priming, and the classical and more used housekeeping gene, the glyceraldehyde 3-phosphate dehydrogenase (*GAPDH*), could not be a good reference gene with which to compare LPS-primed macrophages, and other housekeeping genes, such as the hypoxanthine phosphoribosyltransferase 1 (*HPRT1*), must be considered.

Data from the measurement of gene expression can be validated or supported by assessing protein expression by, for example, Western blot (as will be reviewed latter). This would be valuable in circumstances that the mRNA is not stable enough to produce a high increase in the protein amount, and demonstrating that a specific inflammasome inhibitor does not affect the protein expression is valuable information.

Gene expression is a technique that could be scaled up, but is not recommended for initial screening when specific inflammasome inhibitors are of interest, since potential positive compounds have little chances to block the inflammasome activation, but rather interfering with its cellular expression.

### 4.2. Cytokine Release

Upon inflammasome activation, the activation of caspase-1 induces the release of the processed form of the pro-inflammatory cytokines IL-1β and IL-18. Determination of these cytokines in cell supernatants (usually from macrophages) after in vitro models of inflammasome activation, or in the serum or peritoneal lavage from different in vivo disease models is commonly a readout of inflammasome activation where specific inhibitors can be tested. Specific activation protocols for the different inflammasomes will allow the testing of inhibitors against that particular inflammasome, but the use of animals or cells expressing inflammasome sensors with gain of function mutations are an additional model to validate the specificity of such molecules.

#### 4.2.1. Enzyme-Linked Immunosorbent Assay (ELISA)

One of the most extended techniques to detect cytokines is the ELISA determination. ELISA gives information about the concentration of a specific cytokine in cell supernatants and biological fluids. Molecules affecting inflammasome activation will result in a decreased release of IL-1β or IL-18. Although antibodies used in these ELISA have been optimized to detect the mature processed form of IL-1β or IL-18 (the C-terminal) versus the full pro-form of these cytokines, they also detect the pro-form of these cytokines. Therefore, lysis of primed cells will result in a positive ELISA signal for these cytokines, and compounds protecting cell death, would result in a decrease of the detected signal. So, validation of the inhibition of pro-IL-1β or pro-IL-18 maturation should also be confirmed by Western blot (see Section 4.2.2). Alternatives to measure release of IL-1β or IL-18 are to measure other proteins such as ASC, high motility group box 1 (HMGB1), or inflammasome sensors that can be released during pyroptosis upon inflammasome activation [78]. These molecules do not depend on processing, but their release depends on late phase of pyroptosis, however, specific ELISAs for these molecules are less developed than for IL-1β or IL-18.

ELISA could be easily scaled up to do initial screening of compounds targeting specific inflammasomes (especially if they are added after priming), but detected molecules using this technique could be blocking other steps upstream and downstream of the inflammasome, such as the signaling to activate the inflammasome or caspase-1 activity or pyroptosis. Therefore, compounds blocking the release of IL-1β or IL-18 found by ELISA should be evaluated using other techniques to elucidate if they directly target the target inflammasome.

#### 4.2.2. Western Blot

This technique could confirm at the protein level the effect of different specific inflammasome inhibitors on the expression of different inflammasome components and to analyze the processing of IL-1β, IL-18, or GSDMD after inflammasome induction. The processing of these proteins can be blocked or reduced if a specific inhibitor against inflammasomes has been added in the in vitro inflammasome activation assay or administrated in an in vivo model of disease. However, the blocking of the processing of these targets should be interpreted with caution, since if the inhibitor affects caspase-1 activity, the readout of this assay will be the same as if the inhibitor blocks the inflammasome activation. So, the results from Western blots must be accompanied by others techniques to check the mechanism of action of the small molecule.

### 4.3. Caspase-1 Activity Determination

Caspase-1 activity is a common readout for different possible inflammasome blocking compounds. However, molecules blocking IL-1β release after inflammasome activation, specifically bind to reduced cysteine (Cys-SH) residues within the active site of caspase-1, blocking its enzymatic activity instead of the inflammasome. In fact, different compounds blocking GSDMD-induced pyroptosis by oxidizing GSDMD cysteine residues, also block caspase-1 activity [79,80,81]. To check this, caspase-1 activity can be directly determined using a fluorometric technique that is based on the cleavage of a tetra-peptide substrate YVAD linked to 7-amino-4-trifluoromethyl coumarin (AFC). Once the substrate is cleavage, the AFC group emits a yellow-green fluorescence that can be quantified by a specific fluorescence reader. This fluorescent substrate could be processed by recombinant caspase-1 (as a control) or by cell extracts where caspase-1 has been activated. This technique has been used to verify that a molecule targeting the NLRP3 inflammasome was not directly inhibiting caspase-1 [82].

### 4.4. Determination of Pyroptotic Cell Death

#### 4.4.1. Lactate Dehydrogenase Measurements

Inflammasome activation will induce pyroptotic cell death by the cleavage of GSDMD and the subsequent formation of GSDMD pores on the plasma membrane that allow the direct release of mature IL-1β or IL-18. If GSDMD pores are not properly controlled, the cell swells and the plasma membrane collapses by the action of the ill-characterized nerve injury-induced protein 1 (NINJ1), releasing bigger molecules from the cell as lactate dehydrogenase (LDH) [83]. LDH is a tetrameric enzyme that does not permeate through GSDMD pores, and therefore its release occurs during the lytic-pyroptotic phase. The combination of LDH determination in cell supernatants together with IL-1β or IL-18 determination is highly interesting, because it could potentially identify molecules affecting IL-1β release and not pyroptotic cell lysis. Since LDH is an enzyme, its detection is based on the determination of its catalytic activity by colorimetric or fluorogenic methods that can be easily scaled up. When using this method, a control experiment needs to confirm that the molecules inducing a decrease of LDH release after inflammasome activation are not directly blocking the LDH enzymatic activity. Positive molecules that report a decrease of LDH release after inflammasome activation could be acting upstream or downstream inflammasome activation and do not need to directly target inflammasome formation. Therefore, it is a good assay to screen selected collections of small molecules as it can be easily scaled up, but maybe too risky for an initial high throughput screening assay.

#### 4.4.2. Plasma Membrane Permeabilization

The measurement of pyroptotic cell death can be also monitored using small fluorescent dyes that permeate through GSDMs pores, but not through intact membranes. Upon uptake, these molecules bind to intracellular DNA and will become fluorescent. An inflammasome-inhibiting molecule should reduce fluorescence after inflammasome activation when compared to cells not treated with the inhibitor. There are many different DNA-binding fluorescent dyes that are impermeant to intact plasma membrane, among them we find YO-PRO^TM^-1 iodide, YOYO^TM^-1 iodide, propidium iodide, or ethidium bromide. The uptake of these molecules could be monitored by fluorescence microscopy, fluorescent plate readers, image stream systems, or flow cytometry.

#### 4.4.3. Microscopic Evaluation of Pyroptotic Cells

Visualization of the pyroptotic cells by bright field microscope can be helpful and complement to the assays of dye uptake explained above. Pyroptotic cells present a specific morphology with condensed nuclei and the presence of a big bleb. Bright field images comparing cells after inflammasome activation in the presence/absence of different inflammasome inhibitors could reveal if the compound is affecting pyroptosis. However, this technique does not allow scale up and could be used as a tool to explore the morphology of the cells treated with the small molecule in use.

#### 4.4.4. Measurement of Intracellular Content Release

During pyroptosis, the intracellular content of cells is released to the extracellular media. Besides proteins, the K^+^ ion is quickly released, and the measurement of intracellular K^+^ by coupled plasma optical emission spectrometry after inflammasome stimulation in the presence or absence of blocking molecules, can also support for inflammasome blocking. When measuring K^+^ efflux it has to be taken into account that different NLRP3 activators are strong inducers of intracellular K^+^ depletion, such as the K^+^ ionophore nigericin or the activation of the purinergic receptor P2X7 by Adenosin-5′-triphosphate (ATP). Therefore, in the case of NLRP3 inflammasome, this technique could be used to check the ability of different NLRP3 inhibitors (CY-09 or MCC950) to block its activation step by preventing intracellular K^+^ decrease [84,85]. This technique is not recommended for a screening method, as it is not an easily scalable technique and the results could confuse the signaling to activate NLRP3.

### 4.5. Biochemical Binding Assays

The specific binding of a small inhibitory molecule to the inflammasome is an important feature that should be analyzed to validate its specific target. This characteristic is one of the more important ones to determine for a specific inhibitor.

#### 4.5.1. Pull-Down Assays

Pull-down assays are widely used for a first approach to know whether a molecule can bind directly to a specific inflammasome target. One of the most extended approaches used is when the molecule is conjugated to a specific tag (as biotin) and a recombinant inflammasome sensor protein fussed to another tag (as Flag) are mixed together. If good antibodies working in Western blot exist for the studied inflammasome, cells endogenously expressing the inflammasome sensor could be used. Then a pull-down assay against the tagged-molecule followed by a Western blot for the inflammasome sensor protein will allow us to know if there is an interaction among them. Negative controls should include pull-down with other inflammasome sensor proteins or components implicated in the inflammasome formation. On the other hand, the inhibitory capacity of the tagged inhibitor should be analyzed, as some tags could result in inefficient inhibitors.

The use of recombinant inflammasome sensors will allow us to perform different truncations and mutations to know which part of the protein binds to the inhibitor. An example of this technique is reported by Jiang et al. [84] where a biotin-CY-09 was used to perform a pull-down assay of NLRP3.

This technique requires that the interaction between the inhibitor and the inflammasome sensor should be strong enough or non-reversible to avoid loss of interaction among the different washes that this technique requires. Moreover, pull-down assay is a quite complex technique that would not be suitable for the screening of different compounds, but rather to confirm interaction of selected compounds.

#### 4.5.2. Drug Affinity Responsive Target Stability Technique

Another technique that can give information about the direct binding of a small molecule to a specific inflammasome sensor is the drug affinity responsive target stability (DARTS) assay. DARTS relies on the protection against proteolysis that a small molecule can give to the target protein due to the direct interaction that stabilizes the structure of the protein. This technique has some advantages over pull-down assays, as cell extracts can be used and purified recombinant proteins (which could have altered structures compared to native proteins) are not required. The specific interaction can be analyzed using different truncations of the target inflammasome sensor protein that will allow us to specifically identify the binding domain. This technique was used to identify the direct binding of MCC950 to NLRP3 [85].

#### 4.5.3. Surface Plasma Resonance Technique

The surface plasma resonance (SPR) assay not only gives information about the binding affinity of the inhibitor with the inflammasome sensor protein, but also determines the kinetics, thermodynamics aggregation, and poor solubility. The SPR assay is commonly used for the optimization and characterization of the inhibitor molecule, but can be also used as a screening tool. An example of SPR was performed in the characterization of the NLRP3 inhibitor MCC950 [86].

#### 4.5.4. Chemoproteomic Strategies

These techniques can be used to check the molecular target of a specific inhibitor. The photoaffinity labeling (PAL) is used to study the proteins-ligand interactions, which can help to identify unknown targets of ligands, the protein structure, and binding sites in proteins [87]. The strategy is based on the use of a chemical probe that can covalently bind to the inhibitor in response to light activation. For this purpose, the presence of a photoactive group is required, which reacts by binding to the nearest molecule after the irradiation. The photo-group has to include some specific features as stability in the dark and daylight, little binding steric interferences, and must have an optimal activation with wavelengths that should minimally damage the biological target. The most common photo-groups are phenylazides, phenyldiazirinas, and benzophenonas.

PAL requires time to allow the association between the photo-groups and targets, and when live cells are used, these are lysed, and click chemistry conjugation with the reporter tag moiety is performed. The binding can be visualized against the specific tag using a Western blot. PAL has been used to study the specific interaction of MCC950 to the NACHT domain of NLRP3 [88]. The specific characteristics that the photo-group has to include are difficult to obtain in all at one particle, so this technique could be difficult to perform and is not a good technique to scale up.

#### 4.5.5. Microscale Thermophoresis

Microscale thermophoresis (MST) assays are used to measure the interactions between an inhibitor and an inflammasome sensor in solution directly without the need of any immobilization surface. MST can be used to detect the binding between any kind of biomolecules including proteins and small molecules as for example specific inhibitors [84]. It is based on the quantifiable detection of a fluorescent variation with the temperature. MST can be helpful to go deeper in the role and characteristics of a selected molecule, but not to perform a screening of a wide range of compounds.

### 4.6. Measurement of ATPase Activity

The NLR inflammasome sensors present ATP hydrolytic activity required for inflammasome assembly [89]. Measurement of the ATPase activity of the NLRs in the presence or absence of inhibitors not only gives information about the direct inhibitory effect, but is also an approach to know the mechanism of action of the inhibitor. The conversion of ATP into Adenosis diphosphate (ADP) by the NLR could be determined using bioluminescent assays [82,84,90]. These measurements could be applicated to small and medium screening assays to differentiate the efficacy of different compound series.

### 4.7. Determination of Inflammasome Oligomerization

Inflammasome sensor molecules usually homo-oligomerize to produce active multi-protein complexes that recruit the adaptor ASC protein. An ideal inflammasome inhibitor will directly bind the inflammasome sensor and will prevent the formation of homo-oligomers. The determination of inflammasome oligomerization can be used to determine the inhibitory actions of different molecules.

#### 4.7.1. Ultracentrifugation Separation Technique

Cells where the desired inflammasome was activated in the presence or absence of different inhibitors can be used to do protein extracts that are separated in soluble and insoluble fractions using ultracentrifugation. Inflammasome oligomers (either the sensor protein or the adaptor ASC protein) are determined in the different fractions by Western blot. This technique is quite complex and time consuming, so it does not allow scale up.

#### 4.7.2. Microscopy Techniques

**Figure 3:**
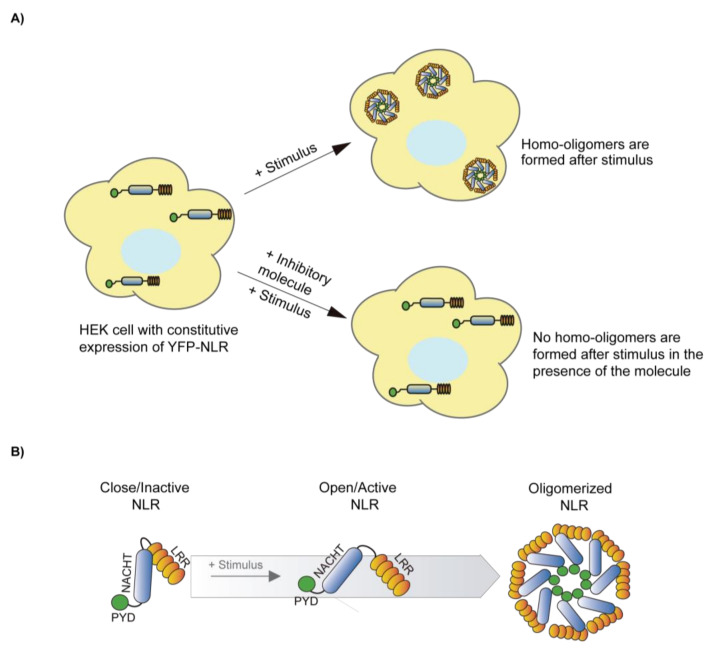
Schematic illustration showing the formation and the inhibition of NLR oligomers. (**A**) Illustration of NLR homo-oligomers formation after a stimulus treatment and the inhibition of homo-oligomers formation in the presence of an inhibitor. If the NLR sensor is fused to a fluorescent reporter protein, the formation of homo-oligomers appears as a puncta across the cytoplasm of the cell. If the cell treatment directly activates the NLR inflammasome, this technique is a reliable screening method to identify specific inflammasome inhibitors. If the signaling leading to inflammasome activation requires different steps, positive compounds could either directly block the inflammasome, or any of these steps. (**B**) Diagram of possible conformational changes of a closed/inactive NLR to an open/active NLR that favor the formation of homo-oligomers.

Microscopic determination of inflammasome oligomerization can be used to perform a fast screening of different compounds. Performing in vitro cellular assays using cells that constitutively express an inflammasome sensor or ASC tagged with a fluorescent protein (as the green fluorescent protein, GFP) will allow the rapid identification of oligomer formation. In the case of NLRs, these proteins are going to be cytosolically distributed in basal conditions and only applying an activation stimulus, so oligomers can be easily detected (Figure 3A). If the treatment with an inhibitor impairs the formation of these oligomers, it probably suggests that either it targets the inflammasome oligomerization or the upstream signaling to activate the inflammasome (Figure 3B) [91]. Alternatively, to activate the inflammasome, the generation of specific mutations in the inflammasome sensor proteins found in patients with autoinflammatory syndromes results in constitutive oligomerization [91]. The NLRP3 inhibitor MCC950 is able to impair constitutive oligomers of NLRP3 with different mutations associated to autoinflammatory syndromes [91]. Washout experiments could be useful to check if the inhibitor is or is not reversible. The formation of ASC oligomers can be also detected by flow cytometry [92], although this technique would easily be implemented in soluble cells, but not in adherence cells.

### 4.8. Bioluminescence Resonance Energy Transfer Assay

**Figure 4:**
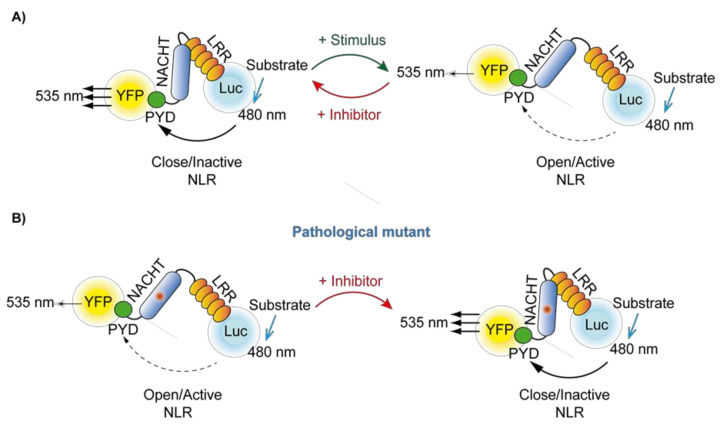
Schematic representation of the Bioluminescent Resonance Energy Transfer (BRET) technique. (**A**) When using NLR tagged with YFP and luciferase (Luc) at both N- and C-terminus, the recorded BRET signal could variate when an inactive/closed conformation changes to an active/open conformation after stimulation. The BRET signal could also change in the presence of an inhibitory molecule. This method could not only be used as a screening assay, as it can be easily scaled up, but also could be used to depict the mechanism of action of inflammasome inhibitors. (**B**) If the NLR BRET sensor depicted in (**A**) includes a pathological mutant, the basal conformation is already in an auto-active state. If the mutant NLR is treated with an inhibitor, the BRET signal will change. Of note, this method will only function if the inhibitor is able to significantly alter the conformation of the mutant NLR.

Bioluminescence resonance energy transfer (BRET) can be used to determine the proximity between a donor protein (luciferase) and an acceptor protein (usually the yellow fluorescent protein, YFP) [93,94]. The donor and the acceptor proteins have to be separated by less than 100 Å to obtain energy transfer, and the BRET signal will depend on the distance between both of them. Luciferase emits light using different substrates with a peak wavelength at 480 nm that is able to excite YFP that now emits at 535 nm. The BRET signal is the ratio among the emission at 480 nm and 535 nm, normalized to the same ratio recorded from the luciferase alone. When expressing inflammasome sensor proteins tagged with YFP and luciferase in cells and induce inflammasome activation, the recording of the BRET signal before and after activation allows us to measure inflammasome oligomerization [95]. Tagging the inflammasome sensor protein with both luciferase and YFP at C- and N-terminal domains will allow us to identify potential changes on the sensor structure during activation that will result in a distance change among the luciferase and YFP (Figure 4A) [91]. The incubation of the cells expressing the inflammasome BRET sensors with inhibitors could result in a change of BRET signal if the inhibitor affects the structure of the inflammasome sensor. For example, NLRP3 auto-active mutants result in a decreased BRET signal, that could be recovered by the addition of the inhibitor MCC950 (Figure 4B) [91].

### 4.9. In Silico Approaches

These studies require specific software and hardware, as well as known structures for the desired inflammasome sensor proteins. Two of the most useful techniques are blind docking and molecular dynamics.

Blind docking assays produce important information about where the inhibitor could bind in the inflammasome sensor protein structure. The specific residues involved in the binding to the inhibitor would be also identified, however, this information should need to be confirmed by performing such specific mutations and test them in different assays. Alternatively, molecular dynamics studies will predict how the binding of an inhibitor to an inflammasome sensor will change during this time. These techniques predicted the probable residues in NLRP3 implicated in MCC950 binding [91].

### 4.10. In Vivo Animal Models

After using different in vitro models to characterize the mechanism of action of different inhibitors over specific inflammasomes, in vivo models are used to study the effects of the inhibitors in a complex pathological scenario. However, animal models of disease activate different signaling pathways and the effect of a specific inflammasome inhibitor could be masked [96].

The crystal-induced kidney or liver fibrosis model in mice by diets rich in oxalate or adenine has been used to demonstrate that MCC950, applicated in an early stage of the pathology, could attenuate serum levels of IL-1β and IL-18, as well as kidney damage [97]. Likewise, the administration of monosodium urate (MSU) crystals triggers an acute inflammatory condition via NLRP3 inflammasome activation mimicking human gout pathology, including joint inflammation and arthritis. Marchetti et al. injected MSU crystal into mouse knee joints and demonstrated that OLT1177, an active β-sulfonyl nitrile compound blocking NLRP3 ATPase activity, reduced inflammation in mice [98].

Another example is the generation of acute colitis with dextran sulfate sodium administration. Perera et al. showed the ability of MCC950 to suppress the activation of infiltrating macrophages in the gut by inhibiting the release of pro-inflammatory cytokines and chemokines [99].

Although there is no NLRC4 specific inhibitor, there are some in vivo models that could be useful to study the efficiency of new inhibitory molecules. Kupz et al. activated NLRC4 by *Salmonella*-derived flagellin to study the response of NK cells to *Salmonella typhimurium* infection [100]. Also, Li et al. used bacterial flagellin to activate NLRC4 to study the effects of flagellin and its mutants lacking the ability to activate TLR5 and NLRC4 alone or in combination on the adaptive immune responses against flagellin [101].

These mentioned models are a representative example of how in vivo models could be used to study the action of an inflammasome inhibitory molecule in specific pathology. Although mouse models are excellent to check the action of a molecule in a specific pathology, other animal models such as zebra fish allow us to quickly screen a library of compounds [102].

## 5. Auto-Active Inflammasomes as a Target for Small Molecules

As previously stated, there are different autoinflammatory diseases characterized by gain-of-function mutations within different genes coding for inflammasome sensor proteins. Most of these syndromes cause an overproduction of IL-1β. There are in vivo animal models carrying different of these conserved mutations to study possible therapeutic treatments that will improve patient’s quality of life, so these models can help to obtain important information about how a new inflammasome inhibitory molecule can affect these auto-active inflammasomes.

Familial Mediterranean fever (FMF) is caused by mutations in the *MEFV* gene, which codifies for the Pyrin inflammasome. The most common mutations in these patients that suffer from FMF are M680I, M694V, M694I, V726A, and E148Q. In 2011, Chae et al. designed knock-in in vivo mouse models with the M680I, M694V, and V726A mutations. They demonstrated that the release of IL-1β was dependent on the mutated Pyrin inflammasome and not on other inflammasomes like NLRP3 [76]. In another study, Sharma et al., developed knock-in mice with the V726A mutation of Pyrin. They demonstrated that the inflammatory process in pathological conditions came from caspase-1 and IL-1β, not from IL-1 β or caspase-8 [103]. The knock-in V726A mouse backcrossed with the *Gsdmd*^−/−^ demonstrated that the reduction of pyroptosis improved the pathological condition [104]. Auto-active mutations in the *NLRP3* gene leads to the CAPS inherited disorders [48]. Coll et al. studied the potential therapeutic action of MCC950 in a mouse model of CAPS with the mutation A350V in NLRP3 [85]. There are only a few studies using mutant NLRC4, all of them using in vitro cell cultures of human cells [105,106]. The design of new in vivo models with different mutants of NLRC4 could be of special interest in the study of the effect of new inhibitory drugs that could be generated in the future for this specific inflammasome.

## 6. Synthetic Molecules Inhibitors of the Inflammasome

Due to the (patho)physiological importance of inflammasome activation, different small molecules have been synthetized to control its activation. Some of them were initially produced for a different purpose, but later their activity inhibiting the inflammasome was described. To date, most of the inhibitors have been developed against the NLRP3 inflammasome [6,48]. It should not be forgotten that, although other inflammasomes may not be as attractive as NLPR3, the NLRC4, NLRP6, Pyrin, or AIM2 inflammasomes are implicated in different pathophysiological states. In this section, the different molecules developed as inflammasome inhibitors are summarized.

### 6.1. Sulfonylureas

**Figure 5:**
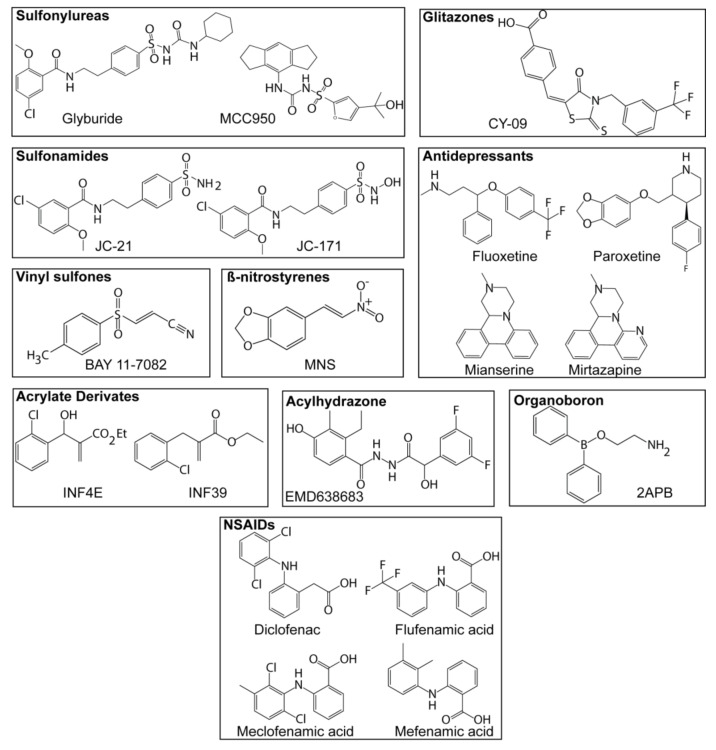
Chemical structure of different NLRP3 inflammasome inhibitors. Chemical structure of different sulfonylureas, sulfonamides, vinylsulfones, β-nitrostyrenes, acrylate derivates, glitazones, antidepressants, acylhydrazones, organoboron, and nonsteroidal anti-inflammatory drugs (NSAIDs) with proven activity as NLRP3 inflammasome inhibitors.

Nowadays sulfonylureas have a great importance as NLRP3 inflammasome inhibitors (Figure 5). Glyburide was one of the first NLRP3 inflammasome inhibitors described, although the first use of this compound was not related with the inflammasome. It has been demonstrated that Glyburide can inhibit NLRP3 inflammasome, but not NLRC4 or AIM2 [107]. Pfizer developed several sulfonylureas with the ability to block IL-1β release [108,109], one of them, the CP-456,773 was later found as a specific NLRP3 inflammasome inhibitor and was renamed to MCC950 [85]. This small molecule is a great starting point for the synthesis of new modified sulfonylureas due to the specificity that shows blocking the NLRP3 inflammasome. Recent studies have described the mechanism of action of MCC950 as binding to the Walker B site of NLRP3 and impairing its ATPase activity [86,91].

### 6.2. Sulfonamides

The most used sulfonamide to block NLRP3 is a synthetic precursor of Glyburide called JC-21 (Figure 5) [110], although JC-21 potency has been improved by the related compound named JC-171 [111]. JC-171 interacts with the allosteric site of NLRP3, which is closer to the ATP binding site, and inhibits the oligomerization of NLRP3 after stimulation [112].

### 6.3. Vinylsulfones

Bay 11-7082 (Figure 5) is a vinylsulfone able to block NLRP3 activation [113], however, this compound can also block NF-κB activation via IKKβ kinase. Parthenolide is another vinylsulfone that blocks caspase-1, and therefore prevents caspase-1 activity in response to the activation of NLRP3, NLRC4, and AIM2 inflammasomes [113].

### 6.4. β-Nitrostyrenes

3,4-methylenedioxy-beta-nitrostyrene (MNS) (Figure 5) interacts with the LRR and NACHT domains of NLRP3, acting as an NLRP3 inhibitor that does not affect NLRC4 or AIM2 activation [114].

### 6.5. Acrylate Derivates

The acrylate derivates named INF4E and INF39 (Figure 5), are potent irreversible NLRP3 inhibitors [82,115], this inhibition seems dependent on a direct interaction of INF39 with NLRP3 and a subsequent reduction of the ATPase activity of NLRP3 [82].

### 6.6. Glitazones

The glitazone CY-09 (Figure 5) has been described as an NLRP3 inhibitor. It have been reported that CY-09 directly binds to the NLRP3 NACHT domain, specifically in the Walker A domain for nucleotide binding, resulting in a blockage of the ATPase activity of NLRP3 and its inhibition [84].

### 6.7. Antidepressants

Drugs as fluoxetine, paroxetine, mianserin, mirtazapine, and agomelotine (Figure 5) have been shown as NLRP3 inhibitors [116], although additional studies about the mechanism of action of these molecules are needed.

### 6.8. Acylhydrazones

The acylhydrazone named EMD638683 (Figure 5) has been reported as an NLRP3 inhibitor when applicated in in vivo animal models of cardiac fibrosis, however, its mechanism of action appears to affect *Nlrp3* and *Il1b* gene expression [117].

### 6.9. Organoboron Derivates

A list of boron compounds derived from 2-aminoethyl diphenyl borinate (2-APB) (Figure 5) without the Ca^2+^ blocking activity, have been described as potent inhibitors of the NLRP3 inflammasome [118]. The pharmacophore for these molecules responsible for the inhibition of NLRP3 is the oxazaborine ring and the electron-withdrawing trichloromethyl (CCl3) group. Structure-activity relationships of the phenyl ring substitution were performed based on some of the organoboron derivates to improve potency and drug-like properties, showing that the lipophicility of the CCl3 group is key to inhibit the NLRP3 inflammasome [119].

### 6.10. Non-Steroidal Anti-Inflammatory Compounds

Four fenamate derivates (diclofenac, flufenamic acid, meclofenamic acid, and mefenamic acid) (Figure 5) affect NLRP3 inflammasome activation. These fenamate derivates affect NLRP3-dependent IL-1β secretion in an Alzheimer’s disease in vivo model, without affecting NLRC4 and AIM2 inflammasomes [120]. These compounds are already being used clinically, which therefore opens the possibility to study the effect of NLRP3 inhibition in relevant clinical settings. However, these compounds also have additional targets such as cyclooxygenase that could synergize with their ability to block NLRP3 as anti-inflammatory therapies.

## 7. Conclusions

Inflammasomes are important signaling molecules of the innate immune response involved in different pathologies. The design of novel small molecules targeting the different inflammasomes has started, with several specific molecules blocking the NLRP3 inflammasome already in clinical trials. However, different experimental techniques are required to depict the molecular mechanism of action of these compounds. The ideal inflammasome inhibitor would need to bind to the inflammasome sensor protein and impair its oligomerization. Different compounds have been shown to target the ATPase activity of NLRP3 by direct association to the nucleotide binding pocket and impairing ATP binding and hydrolysis. Nowadays, compounds targeting other inflammasomes beyond NLRP3 are not described, but the involvement of different inflammasomes in disease suggest their clinical value. Therefore, the different techniques described in this review would allow us not only to do robust compound screening, but also to study the specificity and mechanism of action of novel small identified molecules targeting different inflammasomes.

## Figures and Tables

**Table 1:** PAMPs (pathogen-associated molecular patterns) and DAMPs (damage-associated molecular patterns) responsible for inflammasome activation.

**PAMPs**	Lipopolysaccharide (LPS), Bacterial lipoproteins and lipopeptides, Porins, Peptidoglycan, Lipoteichoic acids, Mannose-rich glycans, Flagellin, Bacterial and viral nucleic acid, Single and double-stranded viral RNA, Glycolipids, Zymosan, lipids from microbial membranes.
**DAMPs**	Adenosin-5′-phosphate (ATP), Adenosine monophosphate (AMP), Adenosine, High mobility group box 1 (HMGB1), Double-stranded DNA, Chromatin, RNA, Monosodium urate, oxidation products, Heat shock proteins, Defensins, β-amyloid, Calcium binding proteins, Mitochondrial DNA, Matrix proteins, Hyaluronic acid, Collagen peptides, Integrins.

**Table 2:** Activators of Different Inflammasomes.

Inflammasome	Activators
NLRP1b	*B. anthracis* lethal toxin, *T. gondii*, Muramyl dipeptide
NLRP3	*C. albicans*, *S. cerevisiae*, *S. aureus*, *L. monocytogenes*, Influenza virus, Sendai virus, Adenovirus, Bacterial pore-forming toxins, Hemozoin, Silica, Asbestos, Ultra violet (U.V.), ATP, Glucose, Monosodium urate (MSU) crystals, Calcium pyrophosphate dehydrate, β-amyloid, Aluminum particles (Alum), Imiquimod, Hyaluronan, ROS, Cholesterol crystals, Cell swelling
NLRC4	Cytosolic Flagellin, Type III secretion system (T3SS) rod protein, T3SS needle complex protein PrgI
NLRP6	Lipoteichoic acid, Gut metabolites, Microbial RNA, LPS
AIM2	dsDNA from DNA viruses or cytosolic bacteria
Pyrin	Bacterial toxins-inducing Rho guanosine triphosphatase (Rho GTPase) inhibition, such as the ones from *C. difficile*, *H. somni*, *V. parahaemolyticus*, or *Y. pestis*

## Data Availability

Not applicable.

## Abbrevations

PAMPs	Pathogen-associated molecular patterns
DAMPs	Damage-associated molecular patterns
HAMPs	Homeostasis-altering molecular processes
NLRs	Nod-Like receptors
ALRs	Absent in melanoma 2-like receptors
ASC	Apoptosis-associated speck-like protein containing a caspase recruitment and activation domain
IL	Interleukin
GSDMD	Gasdermin D
LPS	Lipopolysaccharide
ATP	Adenosin-5′-phosphate
AMP	Adenosine monophosphate
HMGB1	High mobility group box 1
NACHT	Neuronal apoptosis inhibitory protein NAIP, major histocompatibility class II transcription activator CIITA, incompatibility locus protein from Podospora anserina HET-E, and telomerase-associated protein TP1
FIIND	A function to find domain
ROS	Reactive oxygen species
TLR	Toll-like receptor
NF-κB	Nuclear factor kappa B
TNFα	Tumor necrosis factor
NEK7	Never in mitosis gene a (NIMA)-related kinase 7
CAPS	Cryopyrin-associated periodic syndromes
NOMID	Neonatal-onset multisystem inflammatory disease
MWS	Muckle-Wells syndrome
FCAS	Familial cold autoinflammatory syndrome
NAIPs	NLR family of apoptosis inhibitory proteins
MAS	Macrophage activating syndrome
GBP	Guanylate binding proteins
SPRY	Spia and the ryanodine receptor domain
TcdB	Clostridium difficile toxin B
U.V	Ultraviolet
MSU	Monosodium urate
Alum	Aluminum
T3SS	Type III secretion system
qPCR	Quantitative polymerase chain reaction
GAPDH	Glyceraldehyde 3-phosphate dehydrogenase
HPRT1	Hypoxanthine phosphoribosyltransferase 1
ELISA	Enzyme-linked immunosorbent assay
LDH	Lactate dehydrogenase
NINJ1	Nerve-injury-induced protein 1
DARTS	Drug affinity responsive target stability
SPR	Surface plasma resonance
PAL	Photoaffinity labeling
MST	Microscale thermophoresis
ADP	Adenosine diphosphate
GFP	Green fluorescent protein
BRET	Bioluminescence resonance energy transfer
FMF	Familial Mediterranean fever
YFP	Yellow fluorescent protein
